# Sleep quality in first-episode schizophrenia patients

**DOI:** 10.1192/j.eurpsy.2023.2281

**Published:** 2023-07-19

**Authors:** M. Abdellatif, H. Nefzi, O. Abidi, G. Ltaief, R. Kammoun, M. Karoui, F. Ellouze

**Affiliations:** 1 Psychiatry “G” department; 2 Razi Hospital, Manouba, Tunisia

## Abstract

**Introduction:**

Sleep disturbances are commonly observed in schizophrenia and are associated with worse psychotic symptoms and poorer clinical outcomes. This has generated considerable interest in characterizing the relationship between disturbed sleep and schizophrenia.

**Objectives:**

The aim of this study is to assess sleep quality in patients recently diagnosed with schizophrenia that are antipsychotic-naïve and to analyze their association with sociodemographic and clinical data in the same patient group.

**Methods:**

We conducted a cross-sectional, descriptive and analytical study among patients in the psychiatry department "G" at Razi Hospital, over a period of six months. Three scales were used: the Global Assessment of Functioning (GAF) Scale, the Positive and Negative Syndrome Scale (PANSS) and the Pittsburgh Sleep Quality Index (PSQI).

**Results:**

The mean age of patients was 26.8 years and 65% were male. The mean age of onset of the disease in patients included in our study was 24.8 years. The majority of patients (96.3%, n=52) were considered poor sleepers (PSQI threshold value >5). We objectified a negative and statistically significant association between patient functioning (total GAF score) and sleep quality (r= -0.277, p=0.043). The PSQI total score was positively and significantly correlated with the negative scale scores (r=0.315, p=0.021), the general psychopathology scale scores (r=0.411, p=0.002) and the PANSS total score (r=0.378, p=0.005); while no significant association was objectified with scores of the positive scale.

**Conclusions:**

Our study has demonstrated a high prevalence of sleep disorders during the first episode of schizophrenia, as well as their association with the severity of symptoms.

**Disclosure of Interest:**

None Declared

